# Experiences of childbirth care among immigrant and non-immigrant women: a cross-sectional questionnaire study from a hospital in Norway

**DOI:** 10.1186/s12884-023-05725-z

**Published:** 2023-05-27

**Authors:** Kristin Reppen, Lena Henriksen, Berit Schei, Elisabeth Balstad Magnussen, Jennifer Jean Infanti

**Affiliations:** 1 Department of Public Health and Nursing, Norwegian University of Science and Technology, Trondheim, Norway; 2 Department of Nursing and Health Promotion, Oslo Metropolitan University, Oslo, Norway; 3 Division of Obstetrics and Gynecology, St. Olavs University Hospital, Trondheim, Norway; 4 Department of Clinical and Molecular Medicine, Norwegian University of Science and Technology, Trondheim, Norway

**Keywords:** Maternity care, Migrant, Migrant health, Quality of care, Childbirth, Childbirth experience, Perinatal care

## Abstract

**Background:**

Immigrant women have higher risks for poor pregnancy outcomes and unsatisfactory birth experiences than the general population. The mechanisms behind these associations remain largely unknown, but they may result from differential care provided to immigrant women or unsatisfactory interactions with health providers. This study aimed to investigate immigrant and non-immigrant women's experiences of health care during childbirth, particularly assessing two dimensions: perceived general quality of care and attainment of health care needs during childbirth.

**Methods:**

This was a cross-sectional study carried out over 15 months in 2020 and 2021, and data were collected from a self-completed questionnaire. The labour and birth subscale from the Experience of Maternity Care questionnaire was used to assess the primary outcome of care experiences. A total of 680 women completed the questionnaire approximately within two days after birth (mean 2.1 days) at a hospital in Trondheim, in central Norway. The questionnaire was provided in eight languages.

**Results:**

The 680 respondents were classified as immigrants (*n* = 153) and non-immigrants (*n* = 527). Most women rated their quality of care during childbirth as high (91.5%). However, one-quarter of the women (26.6%) reported unmet health care needs during childbirth. Multiparous immigrant women were more likely than multiparous non-immigrant women to report that their health care needs were unmet during childbirth (OR: 3.31, 95% CI: 1.91–5.72, *p* < 0.001, aOR: 2.83, 95% CI: 1.53–5.18, *p* = 0.001). No other significant differences between immigrant versus non-immigrant women were found in subjective ratings of childbirth care experiences. Having a Norwegian-born partner and a high level of Norwegian language skills did not influence the immigrant women's experience of childbirth care.

**Conclusions:**

Our findings indicate that many women feel they receive high-quality health care during childbirth, but a considerable number still report not having their health care needs met. Also, multiparous immigrant women report significantly more unmet health care needs than non-immigrants. Further research is required to assess immigrant women's childbirth experiences and for health care providers to give optimal care, which may need to be tailored to a woman's cultural background and individual expectations.

**Supplementary Information:**

The online version contains supplementary material available at 10.1186/s12884-023-05725-z.

## Background

Immigrant women have higher risks of poor pregnancy and childbirth outcomes, such as preterm delivery, caesarean sections, foetal distress, postpartum bleeding and poorer experiences of care than non-immigrant women [1–6]. Communication barriers, low health literacy, and discrimination are shown to cause negative experiences with care for immigrant women [1, 3, 7–10]. These factors, combined with cultural differences, impede mutual understanding between caregiver and receiver, which is needed for an optimal childbirth experience [1, 11–13].

A negative birth experience may have long-lasting consequences for a woman's mental and physical health [14–17]. It has been associated with postpartum depression, lower self-rated health, recollection of labour pain and symptoms of post-traumatic stress disorder and fear of childbirth, which may be particularly significant for first-time mothers [18–26].

Studies from Canada, the UK, Sweden, the United States and Australia have shown that immigrant women rate the care they receive poorer than non-immigrant women in the same country [1, 3, 27]. However, some studies have found no differences between the migrant and the non-immigrant populations when examining the overall ratings of labour and birth experiences [28–30]. Thus, the results are conflicting.

A recent interview study from Oslo, Norway, showed that immigrant women generally report high satisfaction with maternal health care. Still, refugees report being treated differently due to language barriers or skin colour and religion more often than women migrating for family reunification [31]. Another study from Norway, which was survey-based, indicated no significant difference in satisfaction with maternity health care when comparing immigrants and non-immigrants [32]. While some studies in Norway have included immigrant women in their assessment of the quality of maternity care, the majority of studies have excluded them making it difficult to fully understand their experiences and perspectives [33, 34].

There is an increasing number of immigrants across Europe, including Norway, many of whom are of childbearing age [35, 36]. Trondheim is Norway's third-largest city, and its population is increasingly multicultural [37]. In 2020, 23.5% of births were to non-Norwegian-born mothers [38]. However, it is important to note that women with immigrant background are a heterogeneous group, with diverse health determinants and pregnancy experiences and outcomes. Therefore, it is crucial and timely to assess whether women’s needs are being met regarding labour and childbirth in this region, and in a nuanced manner that considers this diversity.

The main objective of this study was to investigate immigrant and non-immigrant women's experience of childbirth care, assessing two dimensions: perceived quality of care and attainment of health care needs during childbirth. To better understand potential differences in women’s experiences of childbirth care between first-time mothers and those with multiple births, the analysis was stratified by parity. The secondary outcome was to examine the influence of immigrant-related factors on women's rating of childbirth care.

## Methods

### Setting

A cross-sectional design was used, collecting information via a self-completed questionnaire among women approximately two days after birth at St. Olavs University Hospital in Trondheim, Norway. Data were collected between 23 September 2020 to 13 December 2021.

### Participants

All eligible women giving birth during the study period were invited to participate. Women were eligible for participation if they were 18 years and above and had given birth to a healthy newborn. Excluded women were those aged below 18, women who suffered from severe mental or somatic disorders or those who could not give informed consent. Women whose newborns had been transferred to the neonatal intensive care unit were excluded. Women were also excluded if they were unwell during data collection or already participating in another research study at the hospital.

### Data collection

The study questionnaire was administered by four experienced midwives working as project research assistants (RAs). Recruitment took place when the RAs were present, approximately every second or third day per week. The number of women recruited on a specific day was restricted by the RAs' work hours. Therefore, immigrant women were prioritised for study recruitment, and then a random sample of non-immigrant women was invited. When an eligible woman was identified on the list, the RA informed her verbally about the study and provided written study information, a consent form, and the questionnaire in a language of the woman's choice among eight available languages. The women were then instructed to complete the questionnaires in their own time and return them, along with the signed consent forms, to a locked collection box provided at the ward. In three cases, the hospital's telephone interpretation service was engaged to explain the study information in the women's native languages. One questionnaire was completed entirely with the support of telephone interpreters.

### Questionnaire

The use of questionnaires is the most common method to evaluate patient satisfaction with maternity care, and a wide variety of instruments are available for this purpose [39]. The labour and childbirth subscale of the Experience of Maternity Care (EMC) questionnaire, developed and validated in a UK population, was selected to measure care experiences during childbirth in this study. The EMC subscale was chosen for its design and purpose to capture specific and nuanced aspects of diverse women's perceptions of childbirth care [40]. Additionally, items from the Migrant Friendly Maternity Care Questionnaire (MFMCQ) were included in the study questionnaire to assess migrant-specific factors, such as women's languages, length of stay in the host country, and companionship [41]. Background variables and socioeconomic factors were assessed using questions from the Nord-Trøndelag Health Study [42].

The questionnaire was professionally translated into six languages—Arabic, Polish, Dari, Farsi, Tigrinya, and Somali—in addition to Norwegian and English. The selection of these languages reflected the largest groups of immigrant women giving birth in the same hospital in 2019 and the languages most frequently requiring telephone interpretation from the hospital's maternity services. The translated versions of the questionnaire were assessed for cultural and linguistic clarity by representatives of the target population, who suggested minor modifications to improve comprehension.

### Outcome variables

The 12-item EMC labour and birth subscale consist of two subcomponents: seven questions related to perceived quality of care during childbirth and five questions related to specific unmet health needs during childbirth. The quality of care during childbirth subcomponent includes staff communication, individualised care, feelings of safety, confidence, and trust in the health system and staff, while the health care needs subcomponent pertains to the adequacy of staff support and pain management, as well as feelings of involvement in the birth process. The questions are shown in detail in Additional file 1. Quality of care during childbirth accounts for staff communication, individualised care, feelings of safety, confidence and trust in the health system and staff. Health care needs relate to the adequacy of staff support and pain management, and feelings of involvement in the birth process.

The women were asked to score their experiences on a five-point Likert scale using the following options: 'Strongly agree', 'Agree to some extent', 'Neither agree nor disagree', 'Disagree to some extent', and 'Strongly disagree', representing scores from 4 to 0, respectively. A sum score for each subcomponent was made by adding all item responses. A low sum score indicated a low quality of care and more unmet health care needs; conversely, high scores reflected perceived high-quality care and health care needs being met during childbirth. The five items in the unmet health needs subcomponent were addressed in a reversed manner, and the scores had to be inversed before analysis.

The women were categorised as perceiving high-quality care and health care needs being met during childbirth if they had a score above 80% of the maximal score and no answer reporting the least favourable response option (i.e., 'Strongly disagree'). The remaining women were categorised as perceiving low-quality care and experiencing unmet health care needs during childbirth. These criteria were set to capture participants' experiences of poor childbirth care and were applied to both subcomponents.

### Explanatory variables

The questionnaire data were supplemented with information from consenting women's medical records. The supplementary information provided information about the mother's highest completed education level, employment status and current and previous obstetrical history, such as the number of prior deliveries and pregnancy complications of the current pregnancy.

The women were classified as immigrants and non-immigrants based on their self-reported country of birth. If this variable was missing in the study questionnaires, information from the women's medical records was used. The same was done for the partner's country of birth. All persons born outside Norway were considered immigrants. Participants born in Norway to immigrant parents counted as non-immigrants. A recent immigrant was defined as a woman born outside Norway who had lived in Norway for five years or less at the time of data collection. A non-recent immigrant was a woman born outside Norway who had lived in Norway for more than five years. The regions of origin of the women were described using four categories that were previously established in relevant migration studies [32]: Norway; Western Europe, North America, and Oceania; Eastern Europe; and Asia, Turkey, Africa, and South America.

Self-assessed Norwegian language proficiency was based on three questions regarding Norwegian speaking, reading and comprehension: 'How well do you understand/speak/read the Norwegian language?' with the options 'Fluently', 'Well', 'With some difficulty' and 'Not at all'. The questions were combined, and Norwegian language proficiency was categorised into two groups: 'Fluently/Well' and 'With difficulty/Not at all'.

Multiparous women's previous birth experiences were coded into 'previous positive experience' (including 'An entirely positive experience' and 'Mainly a positive experience but with negative elements') and 'previous negative experience' (including 'Mainly a negative experience but with positive elements' and 'An entirely negative experience').

### Statistical analysis

The statistical analysis began with calculating frequencies with percentages for the descriptive statistics. The Pearson's chi-square test was used to assess differences in sociodemographic characteristics between immigrant and non-immigrant women. Mean scores were calculated separately for each question for immigrant women and non-immigrants, as well as for each parity group. The main outcome was then assessed using multivariable logistic regression to investigate immigrant and non-immigrant women's experiences with health care during childbirth. Crude odds ratio (OR) and adjusted odds ratios (aOR), and 95% confidence intervals (CI) were calculated by comparing the immigrant and non-immigrant groups. Adjustments were made for mother’s age, highest completed education, and complications during pregnancy. Analyses were performed separately for primiparous and multiparous women, given the significant influence of giving birth for the first compared to subsequent times on emotions such as fear, uncertainty and insecurity [26]. Self-reported negative prior birth experiences were also controlled for in the multiparous women's analyses. For the second question, a subgroup analysis was performed among the immigrant group. Logistic regression was used to investigate immigrant women’s rating of health care during childbirth relative to variables such as region of origin, length of residency in Norway, having a Norwegian partner, Norwegian language proficiency, and interpretation help during labour and birth. The crude and adjusted odds ratios and 95% confidence intervals were calculated. Adjustments were made for mother’s age, highest completed education, and parity. We did not separate the group by parity due to sample size. All analyses were computed using STATA/MP ver. 17.0, and a *p*-value of < 0.05 was set for statistical significance.

### Ethical considerations

The Regional Committee for Medical and Health Research Ethics in Central Norway (REK Central) approved the study in 2020 (project ID: 31332).

## Results

### Characteristics of the study population

**Fig. 1:**
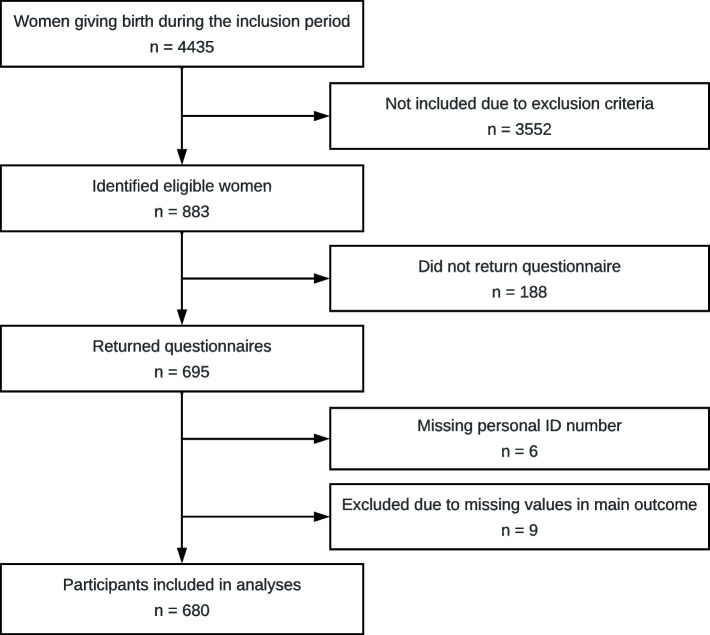
Flow chart of inclusion

Of the 883 women who were identified as both eligible and invited for participation by the RAs, 695 women completed the questionnaire. After removing questionnaires with more than three missing values in the EMC questions (*n* = 9) and excluding participants whose personal ID numbers, needed to assess medical records, were missing (*n* = 6), 680 participants were included in the analyses (Fig. 1). The mean number of days after birth when the women completed the questionnaire was 2.1 days (SD 1.5 days).

Among the participants, 153 (22.5%) were immigrants, and 527 (77.5%) were non-immigrants. The median age for immigrant women was 32.6 years (SD 4.4) and 30.9 years (SD 4.5) for non-immigrant women. There were 47.0% primiparous women in the immigrant group and 52.4% primiparous women in the non-immigrant group (Table 1).

**Table 1:** Sociodemographic and clinical characteristics of participants (*n* = 680)

	Non-immigrant (*n* = 527)		Immigrant (*n* = 153)		All (*n* = 680)	
**Variables**	n	%	n	%	n	%
**Age**						
18–24	38	7.2	7	4.6	45	6.6
25–29	171	32.5	33	21.6	204	30.0
30–34	209	39.7	57	37.3	266	39.1
35 +	109	20.7	56	36.6	165	24.3
**Parity**						
Primipara	276	52.4	72	47.0	348	51.2
Multipara	251	47.6	81	53.0	332	48.8
**Marital status**						
Married/cohabiting/in a relationship	516	97.9	149	97.4	664	97.8
Single/divorced/never married	11	2.1	4	2.6	15	2.2
**Norwegian partner**						
Yes	486	92.2	49	32	535	78.7
No	35	6.6	104	68	139	20.4
No partner	6	1.1	-	-	6	0.9
**Highest completed education**						
Primary/Secondary school	9	1.7	16	10.5	25	3.7
Upper secondary	103	19.5	37	24.2	140	20.6
College/University	415	78.8	98	64.1	513	75.4
No information	-	-	2	1.3	2	0.3
**Employment status** ^a^						
Employed (full-time/part-time/student)	469	89.2	94	61.8	563	83
Unemployed (not working/disability pension/looking for jobs)	22	4.2	35	23	57	8.4
No information	35	6.7	23	15.1	58	8.6
**Household income in NOK**						
< 250 000	3	0.6	15	4	18	2.7
250 000–450 000	24	4.6	24	15.7	48	7.1
451 000–750 000	85	16.1	34	22.2	119	17.5
751 000–1 000 000	167	31.7	23	15	190	27.9
> 1 000 000	238	45.2	34	22.2	272	40
Unknown/Missing information	10	1.9	23	15	33	4.85
**Complications during pregnancy** ^b^						
No	486	92.2	129	84.3	615	90.4
Yes	41	7.8	24	15.7	65	9.6
**Induced labour**						
Yes	139	26.4	32	20.9	171	25.2
No	388	73.6	121	79.1	509	74.9
**Mode of delivery**						
Vaginal delivery non-instrumental	369	70	105	68.6	474	69.7
Vaginal delivery vacuum/forceps	51	9.7	13	8.5	64	9.4
Elective caesarean section	42	8	10	6.5	52	7.7
Emergency caesarean section	65	12.3	25	16.3	90	13.2
**Gestational age in weeks**						
≤ 36 + 6	29	5.5	8	5.2	37	5.4
≥ 37	498	94.5	145	94.8	643	94.6
**Rated care needs during childbirth**						
Unmet health care needs	131	24.9	50	32.7	181	26.6
Health care needs met	396	75.1	103	67.3	499	73.4
**Perceived quality of care during childbirth**						
Low quality of care	36	6.8	22	14.4	58	8.5
High quality of care	491	93.2	131	85.6	622	91.5

*NOK* Norwegian kroner

^a^missing *n* = 2

^b^Bleedings, Hb under 9 g/dL or over 13 g/dL, preeclampsia, and gestational diabetes

Of the 153 immigrant women in our study, forty-three per cent (42.7%) had lived in Norway for five years or less, while fifty-seven per cent (57.3%) had lived in Norway for more than five years. The women came from 59 different countries. The countries are shown in Additional file 2. Regarding Norwegian language skills, 56.4% of the immigrant women reported having 'Fluent/Sufficient' Norwegian skills, and 43.6% rated their skills as 'Low'. One hundred and twenty-one (81.7%) of the immigrant women reported that they always understood the information provided by health care personnel during labour and birth, while 27 women (18.4%) said they understood the information 'sometimes' (Table 2). Of the immigrant women participating in this study, seventy-four (48.4%) participated using a translated questionnaire. Thirty-five women (22.9%) used the English translation, fifteen women (9.8%) used the Polish translation, thirteen (8.5%) used the Arabic translation, five (3.3%) used the Somali translation, five (3.3%) used the Tigrinya translation, and one woman (0.7%) used the Dari translation. The Farsi translation of the questionnaire was not used.

**Table 2:** Characteristics of the immigrant group (*n* = 153)

**Variables**	n	%
**World regions of origin**		
Western Europe, North America and Oceania	35	22.9
Eastern Europe	40	26.1
Asia, Turkey, Africa, and South America	78	51.0
**Length of residency**^a^		
Non-recent immigrants (> 5 years)	71	57.3
Recent immigrants (≤ 5 years)	53	42.7
**Norwegian language proficiency**^b^		
Fluently/Well	84	56.4
With difficulty/Not at all	65	43.6
**Understood the information given by health care professionals **^c^		
Yes, always	121	81.8
Yes, sometimes	27	18.2
Yes, but rarely	-	-
No, never	-	-
**Would have understood the information better in another language **^**d**^		
Yes	60	42.0
No	54	37.8
I don't know	17	11.9
Not relevant	12	8.4
**Did you have someone to help with language interpretation during labour and birth?**^e^		
No, I had no one / Did not need interpretation support	98	66.7
Yes, I had someone for interpretation support	49	33.3

^a^Missing *n* = 29

^b^Missing *n* = 4

^c^Missing *n* = 5

^d^Missing *n* = 10

^e^Missing *n* = 6

### Main outcome

In our study population, many women rated their quality of care during childbirth as high (91.5%). However, one-quarter of the women (26.6%) reported unmet health care needs during childbirth. There was a trend toward primiparous immigrant women rating perceived quality of care lower than the primiparous non-immigrant women. Still, the difference was not significant (OR: 2.08, 95% CI: 0.95–4.52, *p* = 0.064, aOR: 2.15, 95% CI: 0.93–4.96, *p* = 0.072). The analyses showed no statistical difference between immigrant and non-immigrant primiparous women regarding health care needs (Table 3).

**Table 3:** Logistic regression analysis of care experiences between immigrant and non-immigrant women by parity

**Primiparous women (*n* = 348)**						
	OR	95% CI	*P*-value	aOR^a^	95% CI	*P*-value
**Unmet health care needs**						
Non-immigrant	1 (ref)			1 (ref)		
Immigrant	0.63	0.34–1.16	0.140	0.54	0.28–1.03	0.062
**Perceived low quality of care**						
Non-immigrant	1 (ref)			1 (ref)		
Immigrant	2.08	0.95–4.52	0.064	2.15	0.93–4.96	0.072
**Multiparous women (*n* = 332)**						
	OR	95% CI	*P*-value	aOR^b^	95% CI	*P*-value
**Unmet health care needs**						
Non-immigrant	1 (ref)			1 (ref)		
Immigrant	3.31	1.91–5.72	< 0.001	2.82	1.53–5.18	0.001
**Perceived low quality of care**						
Non-immigrant	1 (ref)			1 (ref)		
Immigrant	2.66	1.15–6.12	0.021	2.41	0.94–6.20	0.067

*Ref* Reference

^a^Adjusted for mother's age, highest completed education, and complications during pregnancy

^b^Adjusted for mothers age, highest completed education, complications during pregnancy, and self-reported previous negative birth experience (Missing *n* = 11)

In the group of multiparous women, immigrants were almost three times more likely to report unmet health care needs when compared to the multiparous non-immigrant group (OR: 3.31, 95% CI: 1.91–5.72, *p* < 0.001, aOR: 2.83, 95% CI: 1.53–5.18, *p* = 0.001). Immigrant women reported lower scores for health care needs, especially regarding staff support and being left alone during birth, compared to non-immigrants. The mean scores of each item for immigrant and non-immigrant women, separated by parity, are shown in Additional file 3. The analyses also showed a statistically significant difference in the multiparous group regarding perceived quality of care (OR: 2.66, 95% CI: 1.15–6.12, *p* = 0.021) but fell below the level of significance after controlling for relevant variables (aOR: 2.41, 95% CI: 0.94–6.20, *p* = 0.067) (Table 3).

### Secondary outcome

Our secondary outcome involved assessing the influence of immigrant-related factors on women's rating of childbirth care. Immigrants from Asia, Turkey, Africa, and South America were more likely to report that they experienced unmet health care needs during childbirth (OR: 2.74, 95% CI: 1.10–6.80, *p* = 0.029) when compared to immigrants from Western Europe, North America and Oceania. This difference was not significant after controlling for age, education, and parity. No significant differences in the perceived quality of care or unmet health care needs were found between different subgroups of immigrants in terms of Norwegian language skills, length of residency, and having a Norwegian partner (Table 4).

**Table 4:** Logistic regression analysis of immigrant-related factors associated with unmet health care needs and perceived low quality of care (*n* = 153)

**Unmet health care needs**						
	OR	95% CI	*P*-value	OR^a^	95% CI	*P*-value
Factors						
**World regions of origin**						
Western Europe, North America and Oceania	1 (ref)			1 (ref)		
Eastern Europe	0.71	0.23–2.22	0.564	0.76	0.22–2.55	0.660
Asia, Turkey, Africa, and South America	2.74	1.10–6.80	0.029	2.16	0.80–5.79	0.125
**Length of residency**						
Non-recent immigrants (> 5 years)	1 (ref)			1 (ref)		
Recent immigrants (≤ 5 years)	0.62	0.28–1.35	0.231	0.64	0.266–1.58	0.339
**Partner Norwegian**						
Yes	1 (ref)			1 (ref)		
No	1.52	0.72–3.23	0.267	1.29	0.56–2.95	0.542
**Norwegian language proficiency**						
Fluently/Well	1 (ref)			1 (ref)		
With difficulty/Not at all	0.93	0.46–1.88	0.858	0.72	0.31–1.68	0.460
**Did you have someone to help with language interpretation during labour and birth?**						
No, I had no one / Did not need interpretation support	1 (ref)			1 (ref)		
Yes, I had someone for interpretation support	1.66	0.80–3.44	0.168	1.23	0.54–2.80	0.610
**Perceived low quality of care**						
	OR	95% CI	*P*-value	OR^a^	95% CI	*P*-value
Factors:						
**World regions of origin**						
Western Europe, North America and Oceania	1 (ref)			1 (ref)		
Eastern Europe	1.52	0.33–6.89	0.584	0.98	0.19–4.89	0.985
Asia, Turkey, Africa, and South America	2.33	0.62–8.70	0.207	1.68	0.41–6.78	0.465
**Length of residency**						
Non recent immigrants (> 5 years)	1 (ref)			1 (ref)		
Recent immigrants (≤ 5 years)	2.51	0.85–7.44	0.094	2.61	0.78–8.70	0.117
**Partner Norwegian**						
Yes	1 (ref)			1 (ref)		
No	3.42	0.96–12.19	0.057	2.66	0.71–9.96	0.146
**Norwegian language proficiency**						
Fluently/Well	1 (ref)			1 (ref)		
With difficulty/Not at all	1.67	0.67–4.16	0.266	1.32	0.48–3.58	0.582
**Did you have someone to help with language interpretation during labour and birth?**						
No, I had no one / Did not need interpretation support	1 (ref)			1 (ref)		
Yes, I had someone for interpretation support	1.83	0.73–4.61	0.195	1.59	0.60–4.21	0.350

*ref* Reference

^a^Adjusted for mother's age, highest completed education, and parity

## Discussion

This study found that many women rated their quality of care during childbirth as high (91.5%). However, one-quarter of the women (26.6%) reported unmet health care needs during childbirth. We found that multiparous immigrant women were significantly more likely than multiparous non-immigrant women to report that their health care needs were unmet during childbirth. In terms of quality of care, there was a difference between the groups in the crude analyses, but this difference was not significant after controlling for relevant variables. Regarding immigrant-related factors associated with women's subjective ratings of childbirth care, we found no significant differences related to having a Norwegian-born partner and a certain level of Norwegian language skills.

Our findings show that ninety-one per cent of the women in our study rated their quality of care during childbirth as high. This aligns with other studies from Norway, Germany and Canada, which have shown that even though there are some differences, both immigrant and non-immigrant women are generally positive about their overall maternity care [28, 29, 31]. The positive appraisal of birth experiences found in our study may be due to the way the maternity care system is structured in Norway, which is known to provide safe and evidence-based care [43]. It is nevertheless noteworthy that 26.6% of women reported unmet health care needs during childbirth, which implies scope for improvement to increase attainment of needs during childbirth for both immigrant and non-immigrant women.

Multiparous immigrant women in this study reported unmet health care needs during childbirth more often than their Norwegian counterparts, and showed lower scores, especially in relation to staff support. This may be influenced by these women's previous experiences with the Norwegian health care system. A recent qualitative study of immigrant women's experiences with Norwegian maternity health services concluded that some immigrant women might experience Norway's universal health care system as limiting individual choice and enforcing conformity [44]. In turn, this may cause apprehension or distrust in the system, especially where there are also communication barriers. It may be that health care providers (HCP) perceive multiparous women as more demanding despite having prior knowledge of and experience with childbirth and as a result, may act differently toward them, potentially leading to the women’s perception of poorer quality of care. However, future research is required to explore the specific needs and expectations of multiparous immigrant women in the population, as well as HCP experiences, before making such a conclusion. It may also be that the multiparous women in our study reported unmet health care needs because they were comparing this experience to previous childbirth or to childbirth care from another country, shaping different expectations for childbirth care than for multiparous non-immigrant women. Other studies from high-income countries have shown that a significantly larger proportion of immigrant women is more critical of the care they receive during childbirth than the general population [3, 27, 45]. This may have applied in our study, but most other studies do not separate women by parity as we did, making it challenging to compare results across contexts.

Our finding, showing no significant difference in subjective childbirth experience between immigrant women with or without a Norwegian-born partner contradicts a recent Norwegian study. The authors of that study reported that newly arrived migrant women with a non-Norwegian partner had lower odds of overall care dissatisfaction compared to migrant women with a Norwegian partner [31]. While the influence of having a Norwegian partner on perceptions of the quality of care received by immigrant women during childbirth is unclear, it is possible that having a Norwegian partner may positively influence the woman’s access to information about the healthcare system, language proficiency, and communication with healthcare providers. These factors may contribute to a better understanding of the care received and overall satisfaction with the experience. However, further research is needed to investigate the specific mechanisms through which having a Norwegian partner may influence perceptions of care. This discrepancy in the findings could be due to the differences in our study populations. This study included all immigrants for participation, while the other focused only on newly arrived migrants in Norway.

The same study from Norway also showed that migrant women who rated their Norwegian language comprehension as 'good' or 'with difficulties' had decreased odds of being dissatisfied compared to women who categorised themselves as 'fluent'. At the same time, we did not find these differences in our study. Again, this discrepancy is likely due to the other study's participants only being newly arrived refugees, while our study focused on recent and non-recent immigrants. It is more likely that the larger part of our immigrant participants could speak Norwegian or English adequately to make themselves understood than the newly arrived participants in the other study [27]. Other scientific work emphasises that language and communication barriers between immigrant women and HCP in maternity care are some of the main reasons behind immigrant women's negative birth experiences [1]. This discrepancy between our data and other studies could indicate that we failed to include the most disadvantaged women in terms of language skills in our study, thus, this group's representativeness in the study could be weak. Perhaps the most disadvantaged immigrant women, lacking Norwegian and English language competency and experiencing challenges navigating the Norwegian health care system, were also those least interested in participating in research or struggling the most in expressing or sharing their birth experiences. It is important to mention that the immigrant women in this study, and immigrant women in general, are a heterogeneous group, and failure to acknowledge this diversity may result in producing knowledge that reinforces stereotypes and marginalises some subgroups within the larger group of women with immigrant background.

Most of the immigrants who responded to this study's questionnaire reported that they understood the information given by HCP 'All of the time' and 'Most of the time'. Although almost half of the immigrant women (*N* = 60) reported that they would have understood the information better in another language than it was given in, only two of the 153 immigrant women in our study reported that they had used a professional interpreter during their stay at the maternity ward. We do not know if these numbers implicate that no interpreters were offered or available or simply that very few of the women in our study needed interpreters. Nevertheless, it is important that health care professionals recognise and consider immigrant women's desire and need for professional interpreters during pregnancy or at an early stage of childbirth and that they are informed about their choices and options for interpreters before childbirth. Hospital-based language and intercultural interpretation systems are one option implemented in countries with diverse linguistic and multicultural populations, such as Switzerland [46]. Another solution could be to provide immigrant women with multicultural doulas as birth attendants and guidance and language supporters for navigating a new health care system in a new country [47].

### Strengths and limitations

A strength of this study is that it included many participants, representing a broad diversity of countries and languages in the study population. Another strength is the face-to-face recruitment by trained midwives, which allowed for questions and thorough information and explanations about the questionnaire to the potential participants. Providing study information and the questionnaire in eight different languages, as well as offering the use of professional telephone interpreters, are also strengths of this study. These efforts enabled the recruitment of non-Norwegian-speaking women and women with a lack of English language knowledge. It is possible that immigrant women with stronger Norwegian or English language skills were more likely to participate in the study, as they may have been easier to recruit. This means that our sample may overrepresent immigrant women who are more socially integrated, which could limit the applicability of our findings to those who are less integrated. As this is a cross-sectional study, we are not able to show causal relationships between variables. Also, it is uncertain whether our findings are transferrable to other hospitals in Norway or other countries. Women with severe complications during birth were excluded from this study, which in turn limits the representativeness of a more vulnerable population as well as the generalisability of the study results. In addition, asking women to answer questions about and rate the care given by HCP while still in the institution providing care could have biased the responses; women may have been reluctant to express negative experiences despite information that their answers would only be seen by study researchers and not HCP. Also, the timing of our survey could have introduced biases, as the feeling of relief after birth has been shown to potentially impact and bias a woman's recollection of her birth experience [48].

## Conclusions

The results of this study indicate that women, overall, in the hospital setting are receiving high-quality care. However, the health care needs of some women, especially immigrant women, remain unaddressed, pointing to some shortcomings in the provision of childbirth care. This underscores a continuing imperative to identify interventions for immigrant women in Norway that improve communication and information provision as well as address women's subjective feelings of safety in health care. Negative experiences with health care during childbirth can have long-lasting adverse health effects for mothers and children. Therefore, it is of considerable importance to measure women's perceptions of childbirth care effectively and continually as populations evolve.

## Supplementary Information

**Additional file 1. **Experience of Maternity care questionnaire.

**Additional file 2. **Participants' countries of origin (*n* = 680).

**Additional file 3. **Mean scores of each item for immigrant and non-immigrant women, separated by parity.

## Data Availability

The data supporting this study's findings are available from the corresponding author upon reasonable request. The data are not publicly available because they contain information that could compromise the privacy of research participants.

## Abbreviations

aOR: Adjusted odds ratio
CI: Confidence interval
EMC: Experience of Maternity Care Questionnaire
MFMCQ: Migrant Friendly Maternity Care Questionnaire
OR: Odds ratio
HCP: Health care providers
RAs: Research assistants
