# Supplementary material for: Experiences of childbirth care among immigrant and non-immigrant women: a cross-sectional questionnaire study from a hospital in Norway
Source: BMC Pregnancy Childbirth. 2023 May 27;23:394. doi: 10.1186/s12884-023-05725-z (PMC10223892; doi:10.1186/s12884-023-05725-z)
Supplement: Supplementary file 1 — Additional file 1. Experience of Maternity care questionnaire. [file 12884_2023_5725_MOESM1_ESM.docx]

Experience of Maternity care questionnaire

|  | Strongly disagree | Disagree to some extent | Neither agree nor disagree | Agree to some extent | Strongly agree |
| --- | --- | --- | --- | --- | --- |
| Seven item scale about perceived quality of health care |  |  |  |  |  |
| 30) Staff communicated well with me during labour and birth |  |  |  |  |  |
| 32) Everything was explained to me well during labour and birth |  |  |  |  |  |
| 33) I was treated as an individual by staff |  |  |  |  |  |
| 37) I felt safe in the labour and birth environment |  |  |  |  |  |
| 39) I had confidence and trust in the staff caring for me |  |  |  |  |  |
| 40) I did not mind being looked after by midwives or doctors I had not met before |  |  |  |  |  |
| 41) I had the best possible care during labour and birth |  |  |  |  |  |
|  |  |  |  |  |  |
| Five item scale about unmet health care needs |  |  |  |  |  |
| 31) I needed more staff support during labour and birth |  |  |  |  |  |
| 34) I was not involved enough in decisions about procedures that were carried out (e.g., breaking waters, caesarean section) |  |  |  |  |  |
| 35) Health professionals left me alone more than I would have liked |  |  |  |  |  |
| 36) I felt that my pain relief needs were not managed well |  |  |  |  |  |
| 38) The staff could have done more to help me to feel in control of my labour and birth |  |  |  |  |  |
