# Supplementary material for: Experiences of childbirth care among immigrant and non-immigrant women: a cross-sectional questionnaire study from a hospital in Norway
Source: BMC Pregnancy Childbirth. 2023 May 27;23:394. doi: 10.1186/s12884-023-05725-z (PMC10223892; doi:10.1186/s12884-023-05725-z)
Supplement: Supplementary file 2 — Additional file 2. Participants' countries of origin (n = 680). [file 12884_2023_5725_MOESM2_ESM.docx]

Participants country of origin (n = 680)

| **Country of origin** | **Number of participants per country** |
| --- | --- |
| **AFGHANISTAN** | **1** |
| **ALGERIE** | **1** |
| **BRAZIL** | **1** |
| **CANADA** | **1** |
| **CHILE** | **1** |
| **CHINA** | **8** |
| **COLOMBIA** | **2** |
| **CROATIA** | **2** |
| **CZECH REPUBLIC** | **1** |
| **DENMARK** | **5** |
| **ECUADOR** | **1** |
| **ERITREA** | **7** |
| **ETIOPIA** | **3** |
| **FRANCE** | **5** |
| **GERMANY** | **5** |
| **GREECE** | **2** |
| **HONG KONG** | **1** |
| **HUNGARY** | **1** |
| **INDIA** | **2** |
| **INDONESIA** | **1** |
| **IRAN** | **1** |
| **IRAQ** | **2** |
| **ITALY** | **2** |
| **JAPAN** | **1** |
| **KENYA** | **1** |
| **LATVIA** | **2** |
| **LITHUANIA** | **5** |
| **MALAYSIA** | **1** |
| **MAROCCO** | **1** |
| **MAURITIUS** | **1** |
| **MEXICO** | **1** |
| **MOLDOVA** | **1** |
| **MYANMAR** | **1** |
| **NEPAL** | **2** |
| **NETHERLANDS** | **2** |
| **NICARAGUA** | **1** |
| **NORWAY** | **527** |
| **PHILIPPINES** | **8** |
| **POLAND** | **16** |
| **PORTUGAL** | **1** |
| **ROMANIA** | **3** |
| **RUSSIA** | **3** |
| **SLOVAKIA** | **2** |
| **SOMALIA** | **3** |
| **SOUTH KOREA** | **1** |
| **SPAIN** | **2** |
| **SRI LANKA** | **1** |
| **SUDAN** | **2** |
| **SWEDEN** | **5** |
| **SYRIA** | **10** |
| **TAIWAN** | **1** |
| **TAJIKISTAN** | **1** |
| **TANZANIA** | **1** |
| **THAILAND** | **2** |
| **TURKEY** | **4** |
| **UKRAINE** | **4** |
| **UNITED ARAB EMIRATES** | **1** |
| **UNITED KINGDOM** | **3** |
| **UNITED STATES OF AMERICA** | **2** |
| **VIETNAM** | **1** |
| **Total** | **680** |
