# Supplementary material for: Experiences of childbirth care among immigrant and non-immigrant women: a cross-sectional questionnaire study from a hospital in Norway
Source: BMC Pregnancy Childbirth. 2023 May 27;23:394. doi: 10.1186/s12884-023-05725-z (PMC10223892; doi:10.1186/s12884-023-05725-z)
Supplement: Supplementary file 3 — Additional file 3. Mean scores of each item for immigrant and non-immigrant women, separated by parity. [file 12884_2023_5725_MOESM3_ESM.docx]

Mean scores of each item for immigrant and non-immigrant women, separated by parity

| **Primiparous women** | Observations (n) | Mean | SD |
| --- | --- | --- | --- |
| **Seven item scale about perceived quality of health care** |  |  |  |
| **30) Staff communicated well with me during labour and birth** |  |  |  |
| Non-immigrant | 276 | 4.78 | 0.56 |
| Immigrant | 72 | 4.86 | 0.53 |
| **32) Everything was explained to me well during labour and birth** |  |  |  |
| Non-immigrant | 276 | 4.67 | 0.74 |
| Immigrant | 72 | 4.64 | 0.92 |
| **33) I was treated as an individual by staff** |  |  |  |
| Non-immigrant | 272 | 4.81 | 0.63 |
| Immigrant | 71 | 4.55 | 1.14 |
| **37) I felt safe in the labour and birth environment** |  |  |  |
| Non-immigrant | 276 | 4.51 | 0.93 |
| Immigrant | 72 | 4.76 | 0.72 |
| **39) I had confidence and trust in the staff caring for me** |  |  |  |
| Non-immigrant | 276 | 4.82 | 0.51 |
| Immigrant | 72 | 4.72 | 0.77 |
| **40) I did not mind being looked after by midwives or doctors I had not met before** |  |  |  |
| Non-immigrant | 276 | 4.86 | 0.5 |
| Immigrant | 72 | 4.63 | 0.95 |
| **41) I had the best possible care during labour and birth** |  |  |  |
| Non-immigrant | 276 | 4.68 | 0.68 |
| Immigrant | 72 | 4.75 | 0.74 |
|  |  |  |  |
| **Five item scale about unmet health care needs** |  |  |  |
| **31) I needed more staff support during labour and birth** |  |  |  |
| Non-immigrant | 274 | 4.31 | 1.21 |
| Immigrant | 72 | 4.31 | 1.13 |
| **34) I was not involved enough in decisions about procedures that were carried out (e.g. breaking waters. caesarean section)** |  |  |  |
| Non-immigrant | 276 | 4.40 | 1.12 |
| Immigrant | 71 | 4.44 | 1.16 |
| **35) Health professionals left me alone more than I would have liked** |  |  |  |
| Non-immigrant | 275 | 4.50 | 1.03 |
| Immigrant | 72 | 4.67 | 0.8 |
| **36) I felt that my pain relief needs were not managed well** |  |  |  |
| Non-immigrant | 275 | 4.20 | 1.22 |
| Immigrant | 72 | 4.29 | 1.11 |
| **38) The staff could have done more to help me to feel in control of my labour and birth** |  |  |  |
| Non-immigrant | 275 | 4.26 | 1.18 |
| Immigrant | 72 | 4.47 | 1.08 |

| **Multiparous women** | Observations (n) | Mean | SD |
| --- | --- | --- | --- |
| **Seven item scale about perceived quality of health care** |  |  |  |
| **30) Staff communicated well with me during labour and birth** |  |  |  |
| Non-immigrant | 251 | 4.85 | 0.49 |
| Immigrant | 81 | 4.78 | 0.61 |
| **32) Everything was explained to me well during labour and birth** |  |  |  |
| Non-immigrant | 251 | 4.77 | 0.63 |
| Immigrant | 81 | 4.74 | 0.51 |
| **33) I was treated as an individual by staff** |  |  |  |
| Non-immigrant | 248 | 4.94 | 0.31 |
| Immigrant | 76 | 4.74 | 0.69 |
| **37) I felt safe in the labour and birth environment** |  |  |  |
| Non-immigrant | 251 | 4.63 | 0.84 |
| Immigrant | 81 | 4.72 | 0.675 |
| **39) I had confidence and trust in the staff caring for me** |  |  |  |
| Non-immigrant | 251 | 4.83 | 0.55 |
| Immigrant | 80 | 4.76 | 0.57 |
| **40) I did not mind being looked after by midwives or doctors I had not met before** |  |  |  |
| Non-immigrant | 251 | 4.90 | 0.44 |
| Immigrant | 79 | 4.52 | 1.15 |
| **41) I had the best possible care during labour and birth** |  |  |  |
| Non-immigrant | 251 | 4.72 | 0.66 |
| Immigrant | 81 | 4.72 | 0.8 |
|  |  |  |  |
| **Five item scale about unmet health care needs** |  |  |  |
| **31) I needed more staff support during labour and birth** |  |  |  |
| Non-immigrant | 250 | 4.54 | 1.01 |
| Immigrant | 75 | 4.04 | 1.41 |
| **34) I was not involved enough in decisions about procedures that were carried out (e.g. breaking waters. caesarean section)** | |  |  |
| Non-immigrant | 251 | 4.61 | 0.93 |
| Immigrant | 78 | 3.83 | 1.57 |
| **35) Health professionals left me alone more than I would have liked** |  |  |  |
| Non-immigrant | 250 | 4.67 | 0.88 |
| Immigrant | 81 | 4.30 | 1.37 |
| **36) I felt that my pain relief needs were not managed well** |  |  |  |
| Non-immigrant | 247 | 4.37 | 1.13 |
| Immigrant | 79 | 3.99 | 1.46 |
| **38) The staff could have done more to help me to feel in control of my labour and birth** |  |  |  |
| Non-immigrant | 251 | 4.51 | 0.99 |
| Immigrant | 80 | 4.14 | 1.37 |
